# Enhancing faba bean (*Vicia faba* L.) genome resources

**DOI:** 10.1093/jxb/erx117

**Published:** 2017-04-17

**Authors:** James W. Cooper, Michael H. Wilson, Martijn F. L. Derks, Sandra Smit, Karl J. Kunert, Christopher Cullis, Christine H. Foyer

**Affiliations:** 1 Centre for Plant Sciences, Faculty of Biology, University of Leeds, Leeds LS2 9JT, UK; 2 Bioinformatics Group, Wageningen University, Droevendaalsesteeg 1, 6708PB Wageningen, The Netherlands; 3 Animal Breeding and Genomics, Wageningen University, Droevendaalsesteeg 1, 6708PB Wageningen, The Netherlands; 4 Forestry and Agricultural Biotechnology Institute, Department of Plant Science, University of Pretoria, Hillcrest, Pretoria 0002, South Africa; 5 Department of Biology, Case Western Reserve University, Cleveland, OH 44106-7080, USA

**Keywords:** Illumina sequencing, legumes, mitochondrial genome, plastome, protein security, RNA-seq analysis.

## Abstract

Grain legume improvement is currently impeded by a lack of genomic resources. The paucity of genome information for faba bean can be attributed to the intrinsic difficulties of assembling/annotating its giant (~13 Gb) genome. In order to address this challenge, RNA-sequencing analysis was performed on faba bean (cv. Wizard) leaves. Read alignment to the faba bean reference transcriptome identified 16 300 high quality unigenes. In addition, Illumina paired-end sequencing was used to establish a baseline for genomic information assembly. Genomic reads were assembled *de novo* into contigs with a size range of 50–5000 bp. Over 85% of sequences did not align to known genes, of which ~10% could be aligned to known repetitive genetic elements. Over 26 000 of the reference transcriptome unigenes could be aligned to DNA-sequencing (DNA-seq) reads with high confidence. Moreover, this comparison identified 56 668 potential splice points in all identified unigenes. Sequence length data were extended at 461 putative loci through alignment of DNA-seq contigs to full-length, publicly available linkage marker sequences. Reads also yielded coverages of 3466× and 650× for the chloroplast and mitochondrial genomes, respectively. Inter- and intraspecies organelle genome comparisons established core legume organelle gene sets, and revealed polymorphic regions of faba bean organelle genomes.

## Highlight

Combining RNA-sequencing analysis and illumina paired-end sequencing, we assembled genomic information for faba bean, extending the coverage of reads, as well as mapping organellar genome sequence variation.

## Introduction

Grain legumes (pulses) such as soybean are second only to cereals in terms of economic and nutritional value (O’Rourke *et al.*, 2014). It is now becoming increasingly recognized that grain legumes have advantages over cereals, both in agriculture and in the human diet (Foyer *et al.*, 2016). Pulses are rich in protein, starch, fibre, and other essential nutrients required in the human diet, and they are also valuable in the production of foodstuffs and animal feed. The protein content of pulses greatly exceeds that of cereals, and their amino acid composition is complementary (Boye *et al.*, 2010; Roy *et al.*, 2010). The fixation of atmospheric nitrogen in leguminous plants (Leguminosae), which occurs through the symbiotic union with soil bacteria (Barton *et al.*, 2014; Lassaletta *et al.*, 2014), is used as a natural means of soil nitrogen fertilization in many parts of the world. The increasing organic market, in which farmers rely on legumes to provide much of the nitrogen needed to support other crops, would favour intensification of legume-based agriculture. Although the global demand for legumes for bioenergy purposes as well as food and feed is increasing (Foyer *et al.*, 2016), the yields of many legumes are currently unstable (Cernay *et al.*, 2015). Crucially, the essential underpinning genetic improvement of many potentially important legumes such as faba beans (*Vicia faba*) is currently limited by a lack of genetic resources. Despite some advances, the development of gene-based resources in legumes such as faba bean has not kept pace with cereal crops (O’Rourke *et al.*, 2014; Foyer *et al.*, 2016).

According to FAOSTAT (2016), faba beans, also called broad beans, are the fourth most widely grown cool season legume after pea (*Pisum sativum*), chickpea (*Cicer arietinum*), and lentil (*Lens culinaris*). They have a protein content that is higher than that of many other common food legumes (Griffiths and Lawes, 1978; Burstin *et al.*, 2011). Moreover, the total grain yield of faba bean is not sacrificed in favour of high seed protein contents. In cool climates, faba beans have advantages over legumes such as soybean because they are adapted to growth under low temperatures. As such, they are well suited to sustainable farming practices, particularly in developing countries that are routinely subject to chilling temperatures (Temesgen *et al.*, 2015). However, faba bean yield remains unstable, as is the case with many other major legumes (Cernay *et al.*, 2015).

The Mendelian inheritance traits for faba bean were first characterized in the 1930s (Erith, 1930); however, faba bean genetics received only intermittent interest in the following years (O’Sullivan and Angra, 2016). In the 1970s, asynaptic mutants were identified, from which a series of trisomic lines was developed and analysed (Sjodin, 1970, 1971). Subsequently, genetic markers could be assigned to physical chromosomes (vaz Patto *et al.*, 1999). In addition, genes controlling rhizobial symbiosis and pigment composition were identified (Duc and Picard, 1986; Duc *et al.*, 1999), with segregating recombinant inbred lines (RILs) being used to determine the Mendelian inheritance of a seed dormancy gene (Ramsay, 1997). Parental germplasm resources from early faba bean research are still available (O’Sullivan and Angra, 2016).

Use of RILs and molecular markers has allowed the production of high resolution linkage maps and the identification of quantitative trait loci (QTLs) (Torres *et al.*, 2010; O’Sullivan and Angra, 2016). The high resolution linkage-based sequences have been used to produce maps that are syntentic with other legumes (Webb *et al.*, 2016). For example, in a map of 687 *V. faba* single nucleotide polymorphism (SNP)-based linkage markers, each was matched to an orthologue in *Medicago truncatula* and assigned a linkage group, analogous to one of the six faba bean chromosomes, allowing a putative alignment of *M. truncatula* genomic regions and sequences to corresponding *V. faba* chromosomes (Webb *et al.*, 2016).

Although faba bean transcriptome data have increased in recent years, surprisingly little DNA sequence data are available in public databases (Ray and Georges, 2010; Kaur *et al.*, 2012; Arun-Chinnappa and McCurdy, 2015; Zhang *et al.*, 2015; O’Sullivan and Angra, 2016). The only public data set of genomic DNA sequences was reported by Yang *et al.* (2012), who performed 454 sequencing on the pooled genomic DNA of 247 accessions in order to identify simple sequence repeat (SSR) markers. The current lack of publicly available genomic sequence data for faba bean may be attributed to the intrinsic difficulties of assembling and annotating the giant (~13 Gb) genome. However, the faba bean mitochondrial genome, which was annotated using plasmid/fosmid libraries, shows extensive repetitive content and a low degree of homology to other sequenced mitochondrial genomes (Negruck, 2013). A study on the inverted-repeat-lacking clade of legumes showed that *L. culinaris* and *V. faba* have plastomes that are smaller in size than other chloroplast genomes, but they have similar levels (6.7%) of repetitive DNA (Sabir *et al.*, 2014). However, a blastn analysis of all intergenic spacer (IGS) regions from the sequenced plastomes failed to produce any hits for the *V. faba* sequence in the nucleotide database in GenBank (Sabir *et al.*, 2014). In the following analysis, genomic and transcriptome sequencing were performed on faba bean, providing essential underpinning foundation data that can be used to improve gene identification, which is a prerequisite for future studies on the improvement of key traits of agronomic importance.

## Materials and methods

The faba bean seeds (*V. faba* cultivar Wizard) used in the following studies were provided by Wherry and Sons Ltd, Rippingale, Lincolnshire, UK. Seeds were sown and plants were self-pollinated in the University of Leeds glasshouse facilities to produce first-generation inbred seeds, which were used in all the following experiments.

### Chromosome identification

Faba bean seeds were imbibed for 3 d until germination. Germinated seeds were stored at 4 ºC for a 24 h period to allow cold-induced metaphase synchronization. The root apex was then excised, fixed, and stained for DNA, followed by chromosome imaging under a light microscope.

### DNA extraction, sequencing, and assembly

Faba bean seeds were imbibed for 48 h in distilled water and the embryonic axes were excised. DNA was extracted from excised embryos using the Qiagen DNeasy plant mini kit (Qiagen Ltd, Manchester, UK). Extracted DNA was quantified by nanodrop, and sequencing was performed at the Leeds Institute of Molecular Medicine (University of Leeds, UK). An Illumina HiSeq2500 platform (Illumina, 2014) was used, running a single paired-end library on two flow cell lanes. FastQC analysis (Arun-Chinnappa and McCurdy, 2015) was performed on raw sequenced reads, and ‘Trim Galore!’ (http://www.bioinformatics.babraham.ac.uk/projects/trim_galore/, last accessed 4 April 2017) was used to remove adaptor sequences using an average Phred quality score of 30. Trimmed reads were aligned to both the Cool Season Food Legume *V. faba* transcriptome database (CSFL; Humann *et al.*, 2016), and the *M. truncatula* reference transcriptome (Benedito *et al.*, 2008). ABySS (Simpson *et al.*, 2009) was used for *de novo* paired-end sequence assembly, with an empirically determined *k*-mer size of 32. Alignment against full-length contigs, corresponding to those used by Webb *et al.* (2016) (BioProject PRJNA225873), was then performed using NCBI BLAST+ (v2.5; Camacho *et al.*, 2009). DNA sequence reads for the Wizard cultivar are available at the Sequence Read Archive (SRA; https://www.ncbi.nlm.nih.gov/sra, last accessed 4 April 2017) under the accession numbers SRR5015739 and SRR5015740.

### RNA extraction, sequencing, and assembly

Faba bean (Wizard cultivar) were grown for 24 d in a controlled-environment chamber under a 12 h day/night cycle with an irradiance of 200–250 μmol m^–2^ s^–1^ and day/night temperatures of 25 °C/20 °C, respectively. Leaf discs (2 cm) were taken from the youngest mature leaves of nine plants. Discs from three biological replicates were combined and a single extraction was performed. A total of three RNA samples (representing nine individual plants) were produced. The mRNA samples were sequenced at the Leeds Institute of Molecular Medicine using an Illumina HiSeq 3000 platform producing paired-end 150 bp reads. FastQC analysis (Andrews, 2010) was performed on raw sequenced reads, and ‘Trim Galore!’ was used to remove adaptor sequences using an average Phred quality score of 30. Trimmed mRNA reads were aligned to the CSFL database (Humann *et al.*, 2016) using Bowtie 2.3 (Langmead and Salzberg, 2012). Aligned sequences were analysed using bedtools 2.26.0 Quinlan and Hall, 2010). Trimmed sequence reads are available at the NCBI SRA (https://www.ncbi.nlm.nih.gov/sra, last accessed 4 April 2017) under the BioProject accession PRJNA369531. Single nucleotide variants (SNVs) were identified between RNA alignments and the reference using a samtools (Li, 2011) mpileup-based pipeline.

### Unigene verification

Alignment data files for the RNA sequencing (RNA-seq) versus CSFL and DNA sequencing (DNA-seq) (*de novo* contigs of >1 kbp) were processed using a bedtools-based pipeline to identify contiguous regions of coverage within the CSFL unigenes. The DNA-seq alignments were subsequently separately processed to identify potential splice/breaks in the alignment of unspliced DNA-seq reads versus the spliced RNA reference, as indicated by peaks of bowtie softclips within a 2 bp window.

### Linkage-enhanced genome contigs

Linkage marker sequences were obtained from the BioProject associated with Webb *et al.* (2016), with marker sequences being identified based on work performed by Khazaei *et al.* (2014) and Ellwood *et al.* (2008). Full-length sequences corresponding to these linkage markers were searched with *de novo* contig sequences using NCBI BLAST+.

### Organellar genome comparisons

Chloroplast and mitochondrial reference sequences for *V. faba* and other species were downloaded from NCBI. The DNA-seq reads were aligned to the relevant reference genomes for chloroplasts and mitochondria using Bowtie2 (Langmead and Salzberg, 2012). These were used to align the DNA-seq reads and the relevant reference genome for chloroplasts and mitochondria. Samtools mpileup was used to identify SNVs. A core set of chloroplast and mitochondrial genes was aligned by BLAST between pairs of species, where one or more genes were present in annotated reference sequences from all species. SNVs in DNA-seq reads were visualized using Integrative Genomics Viewer (Thorvaldsdóttir *et al.*, 2013). The effect of SNVs on coding sequence changes was determined using provean (Choi *et al.*, 2012).

## Results

### Genomic imaging and read assembly

**Fig. 1.**
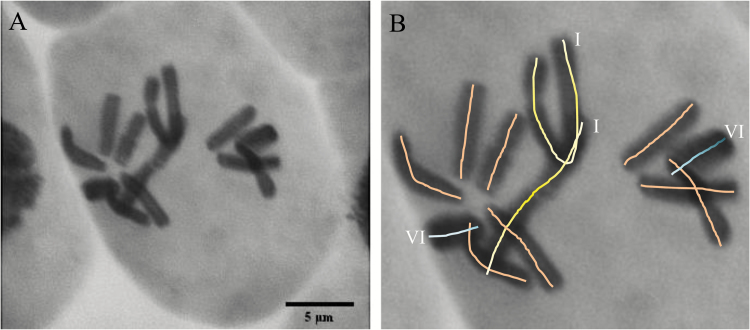
Light microscope image (magnification ×40) of *Vicia faba* chromosomes in metaphase. (A) Feulgen staining of DNA against a FastGreen cytoplasm stain; (B) chromosome labels with roman numerals denote the largest (I) and smallest (VI) chromosome pairs. Scale bars=5 µm.

Cytogenetic analysis showed that the faba bean chromosome number was 2*n*=12 (Fig. 1A). The largest chromosome pair (I) and the smallest chromosome pair (VI) were identified (Fig. 1B), with chromosome I appearing to be considerably larger than the other chromosomes.

DNA was sequenced on an Illumina HiSeq 2500 platform using 100 bp paired-end reads of a single library across two lanes. Following quality trimming, raw reads were aligned to both chloroplast and mitochondrial reference sequences (Table 1). Nearly 13% of reads mapped to the established CSFL reference coding DNA sequence (CDS) of *V. faba* with a total depth of 30×. A small percentage (0.95%) of the reads aligned to the chloroplast reference genome with a coverage of 3466× (Sabir *et al.*, 2014). Similarly, 1.07% of reads aligned to the mitochondrial reference genome with a coverage of 650× (Negruk, 2013). However, 85.1% of reads could not be aligned to either the organellar reference genomes or the cool season food legume database (Table 1; Humann *et al.*, 2016). Of the reads that could not be aligned, only ~10% were identified in a repetitive element database (Bao *et al.*, 2015). The Gypsy class of long terminal repeat (LTR) retrotransposons formed the majority of the repetitive elements, accounting for 3.61% of the total reads. The VicSatellite, LTR (uncharacterized), and Copia class LTR elements comprised 2.49, 2.09, and 0.99% of the genome reads, respectively (Table 2).

**Table 1.** Quality-trimmed genomic read data for DNA extracted from *Vicia faba* (cv. Wizard) detailing read number and coverage, and depth and coverage over the faba bean (CSFL) and *Medicago* v4 reference transcriptomes

	No. of paired reads	Reads accounted for (%)	Coverage
Total number of reads	812 092 660	100.00	6×
Reads aligned to chloroplast	7 712 928	0.95	3466×
Reads aligned to mitochondria	8 657 582	1.07	650×
Reads aligned to faba bean transcriptome database	104 668 765	12.89	30×
Unaligned reads	691 053 385	85.10	<6×

**Table 2.** Quality-trimmed genomic read data aligned against repeat element databases, showing repetitive element identities, the number of paired reads mapped to these repetitive sequences, and the total number of reads expressed as a percentage

Repetitive element	No. of paired reads	Total reads accounted for (%)
LTR/Gypsy	29 358 977	3.615225
VicSatellite	20 274 464	2.496570
LTR (uncharacterized)	16 974 274	2.090189
LTR/Copia	8 090 787	0.996289
rRNA	1 986 302	0.244591
Ogre	1 708 252	0.210352
Other/simple	1 199 173	0.147665
Uncharacterized	1 054 668	0.129870
Mobile element	411 832	0.050712
DNA/En-Spm	332 062	0.040890
Retroelement	92 278	0.011363
Non-LTR	53 835	0.006629
Other	45 904	0.005653
DNA	40 982	0.005046
DNA/MuDR	36 393	0.004481
DNA/hAT	34 576	0.004258
LINE	21 923	0.002700
Satellite	9917	0.001221
DNA/Harbinger	8519	0.001049
tRNA	7049	0.000868
DNA/TcMar	6118	0.000753
DNA/Mite	3643	0.000449
RC/Helitron	1874	0.000231
SINE	883	0.000109
DNA/hAT-Ac	815	0.000100
DNA/Stowaway	344	0.000042
DNA/Tourist	55	0.000007
Other/Centromeric	52	0.000006
DNA/TcMar-Pogo	6	0.000001
Repetitive element total	81 755 957	10.07
Genome uncharacterized	609 297 428	75.03

### Unigene model verification

To enhance the unigenes in the CSFL reference transcriptome, aligned RNA-seq data were generated (96.2 million reads, 14.4 Gbp), aligned, and analysed for breadth and depth of coverage (Table 3). RNA-seq reads showed a large number of patterns of alignment; however, we identified a core set of 16 300 unigenes which showed a single peak of alignments over 95% of the unigene, the majority of which showed an average coverage of at least 10 reads per base. We also observed a similar number with lower breadth, but with a consequent decrease in depth of coverage, as well as decreasing amounts of unigenes showing patterns of alignment peaks.

**Table 3.** Alignment of RNA-seq reads from the Wizard cultivar to the CSFL faba bean transcriptome database Peaks show independent read clustering. Breadth shows the percentage of the unigene model covered, and coverage shows the number of transcripts for which coverages of <10, 10–100, and >100 were obtained. Total shows the total number of unigenes that were present.

No. of peaks	Breadth	Coverage			Total
		<10	10–100	>100	
1	>95%	3067	9169	4064	16 300
	50–95%	9677	5338	1094	16 109
	<50%	4280	47	2	4329
2–3	>95%	326	1515	271	2112
	50–95%	4339	2976	268	7583
	<50%	1198	18	2	1218
4–10	>95%	14	70	5	89
	50–95%	1176	429	17	1622
	<50%	282	8	0	290
	0%				0
>10	>95%	1	0	0	1
	50–95%	15	1	0	16
	<50%	3	0	0	3

In addition, to identify potential genomic structure and putative splice points, we aligned the DNA-seq reads to the CSFL unigenes, and searched the alignment files for indications of localized DNA-seq read alignment breaks (see Supplementary Table S1 at *JXB* online). This uncovered 25 535 unigenes with at least one potential splice site (total 56 668).

### De novo *assembly and linkage map alignment*

**Fig. 2.**
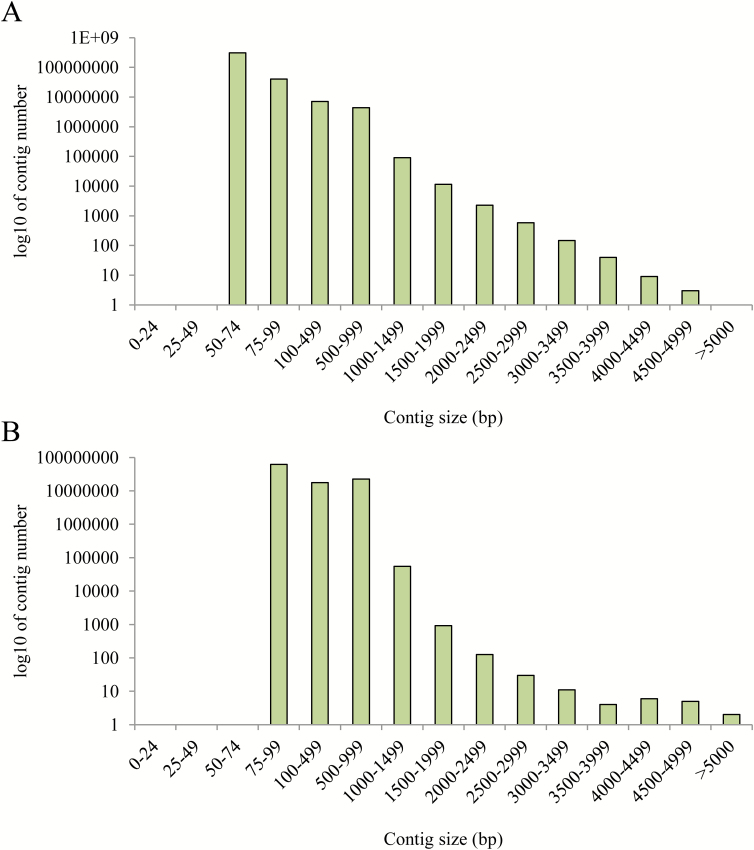
(A) The log_10_ contig abundance over a range of contig sizes, produced from 32 bp *k*-mers; (B) the log_10_ contig abundance over a range of contig sizes, produced from 64 bp *k*-mers.

ABySS assembly of short reads resulted in the generation of >361 million contigs, which varied in length between 50 bp and 5 kb (Fig. 2A, B). The abundance of contigs that were generated was highly dependent on *k*-mer size (Fig. 2A), with 32 bp *k*-mers showing a wider range of contig sizes, together with a higher number of contigs compared with those produced using a *k*-mer size of 64 bp (Fig. 2B). The contigs shown in Fig. 2 were filtered to a subset of 14 682 with a size of ≥1 kbp and aligned against the raw contigs used in the synteny-based SNP linkage map produced by Webb *et al.* (2016). Using a criterion of >95% sequence identity over >95% of a contig, this alignment confirmed the assembled sequence of 94 of the *de novo* contigs, which were smaller than those from Webb *et al.* (2016). The alignment with the existing contigs has also produced an increase in the length of another 461 of these raw loci contigs. A complete list of the syntenic marker-related contigs and the aligned *de novo* contig sequences can be found in Supplementary Table S2. Existing loci contigs were increased by an average of 874 bp (in a range of 55–2995 bp).

### *Single nucleotide polymorphisms in the organellar genome of* Vicia faba

**Fig. 3.**
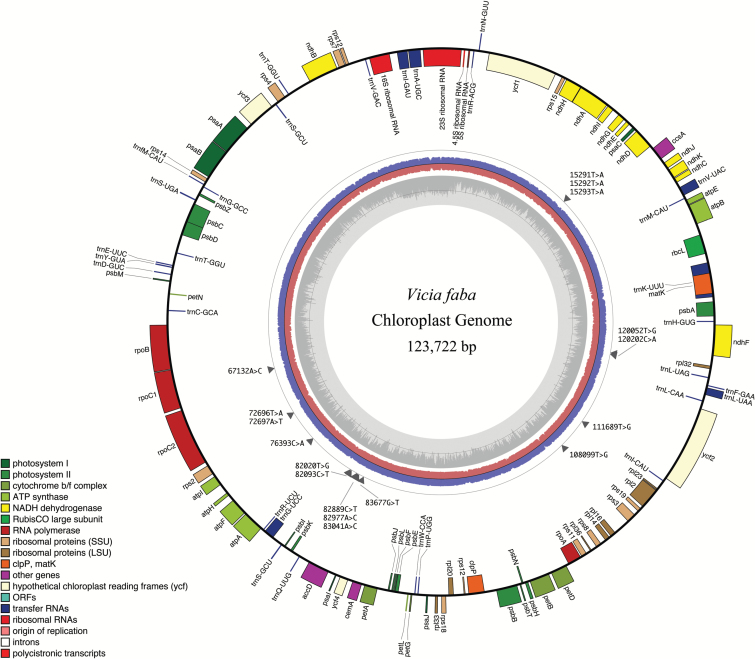
*Vicia faba* chloroplast genome map. The outermost circle shows gene identities and read direction, with outward-facing genes being in the positive direction and inward-facing genes being in the negative direction. SNPs are denoted by grey triangles with the location and nature of polymorphism detailed. Read depth is shown for the forward reads (blue ring) and reverse reads (red ring). GC content (%) is shown by the innermost grey circle, with 50% being denoted by the dark grey line. Gene mapping and annotation were performed in OGDRAW.

Species-level variation within the organellar genomes of *V. faba* raw DNA sequence reads was investigated by mapping against the reference sequences for the chloroplast and mitochondria (Negruk, 2013; Sabir *et al.*, 2014). The locations of gene-based variations within the *V. faba* chloroplast genome (Fig. 3) were obtained by aligning the raw DNA sequence reads to the published faba bean chloroplast sequence (Sabir *et al.*, 2014). While a high level of homology was found between the published reference sequence and the chloroplast genome reported here, a number of SNPs were identified (Table 4). Four genes encoding the RNA polymerase β chain, ATPase subunit I, tRNA Q, and ribosomal protein S3 had a single polymorphism (Table 4). The gene encoding the acetyl co-A carboxylase subunit (*accD*) showed three SNPs. The data in Table 4 show whether the SNPs are synonymous or non-synonymous.

**Table 4.** Single nucleotide polymorphisms in the chloroplast genome of *Vicia faba*, comparing the Wizard cultivar with the reference sequence published by Sabir *et al.* (2014) The amino acid change is provided for non-synonymous mutations including the predicted effect on the protein calculated with Provean. SYN is a synonymous effect.

Map identifier	Gene identity	SNP position	Reference base	Sequenced base	Effect	Provean score
*rpoC1*	RNA polymerase β chain	67 132	A	C	M→L	–1.687
*atpF*	ATPase subunit I	76 393	C	A	–	–
*trnQ-UUG*	tRNA	82 093	G	A	–	–
*accD*	Acetyl co-A carboxylase subunit	82 977	A	C	Y→S	0.775
*accD*	Acetyl co-A carboxylase subunit	83 041	A	C	L→F	–0.77
*accD*	Acetyl co-A carboxylase subunit	83 677	G	T	SYN	–
*rps3*	Ribosomal protein S3	108 099	T	G	K→T	–2.802

**Fig. 4.**
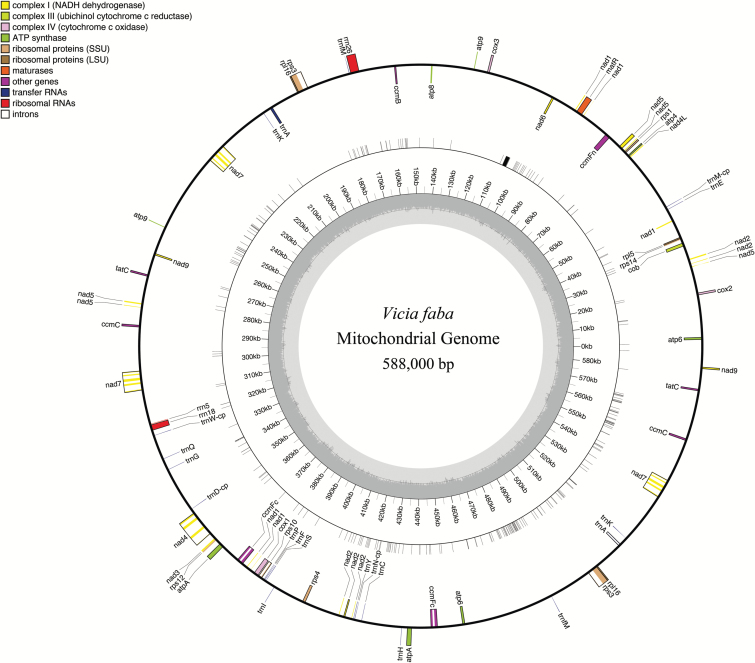
*Vicia faba* mitochondrial genome map. The outermost circle shows gene identities and read direction, with outward-facing genes being in the positive direction and inward-facing genes being in the negative direction. SNPs are denoted by grey bars on the second concentric circle. Base pair positioning is detailed in the third circle. GC content (%) is shown by the innermost grey circle, with 50% being demarked by the dark grey line. The key provides details of mitochondrial gene families. Gene mapping and annotation were performed in OGDRAW.

**Fig. 5.**
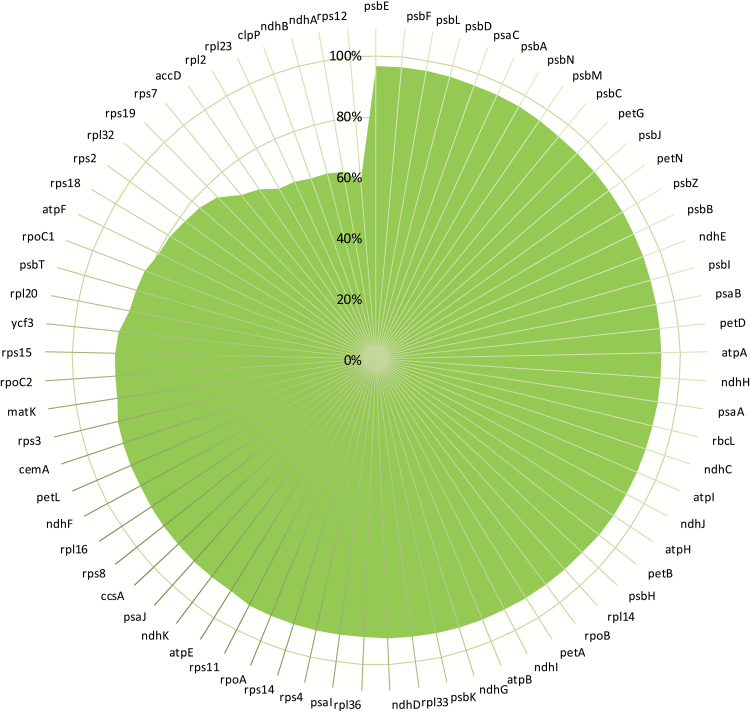
The percentage sequence identity for chloroplast-encoded genes, showing the average data for a 55-way alignment for individual chloroplast genes of 11 species. Gene identities are given in the outermost circle. The scale shows the homology percentage (0–100%). Species comparisons were performed for *Glycine max* (G.m), *Lotus japonicus* (L.j), *Vigna unguiculata* (V.u), *Lens culinaris* (L.c), *Cicer arietinum* (C.a), *Vigna radiata* (V.r), *Pisum sativum* (P.s), *Medicago truncatula* (M.t), *Arachis hypogaea* (A.h), *Phaseolus vulgaris* (P.v), and *Vicia faba* (V.f).

A faba bean mitochondrial genome reference map (Fig. 4) based on genomic read alignment was constructed relative to the cultivar Broad Windsor (Negruk, 2013). While a high level of homology was found between the gene space published by Negruk (2013) and that assembled from raw genomic reads of the Wizard cultivar, seven genes were found to have polymorphisms (Table 5). Analysis of within-species variation for the mitochondrial genome (Fig. 5) revealed a high number of SNPs in the gene coding and intergenic regions. The number of polymorphisms present varied between genes, with the genes encoding 26S rRNA, 18S rRNA, and NADH dehydrogenase subunit 4 having one SNP and the genes encoding maturase R and NADH dehydrogenase subunit 6 having two SNPs. The genes encoding ribosomal protein S14 and cytochrome *c* oxidase subunit II had six SNPs. We checked whether the SNPs found in the chloroplast and mitochondrial genomes will cause an amino acid change (i.e. non-synonymous SNPs). The data in Tables 4 and 5 show that a number of the observed SNPs will change the amino acid and thus they have the potential to have an effect on the functioning of the protein.

**Table 5.** Single nucleotide polymorphisms in the mitochondrial genome of *Vicia faba*, comparing the Wizard cultivar with the reference sequence published by Negruk (2013) The amino acid change is provided for non-synonymous mutations including the predicted effect on the protein calculated with Provean. SYN is a synonymous effect.

Map identifier	Gene identity	SNP position	Reference base	Sequenced base	Effect	Provean score
*rrn26.r01*	26S rRNA	167 803	T	A	–	–
*rrn18.r01*	18S rRNA	321 111	T	G	–	–
*rps14*	Ribosomal protein S14	36 460	T	G	SYN	–
*rps14*	Ribosomal protein S14	36 461	C	G	G→A	–0.404
*rps14*	Ribosomal protein S14	36 462	C	A	G→STOP	–
*rps14*	Ribosomal protein S14	36 463	A	T	SYN	–
*rps14*	Ribosomal protein S14	36 490	G	T	F→L	–1.059
*rps14*	Ribosomal protein S14	36 616	A	G	SYN	–
*nad6*	NADH dehydrogenase subunit 6	101 407	A	T	SYN	–
*nad6*	NADH dehydrogenase subunit 6	101 508	G	T	L→I	–0.026
*nad4.CDS.3*	NADH dehydrogenase subunit 4	361 807	T	G	F→L	4.0
*matR*	Maturase R	90 908	G	T	L→F	1.622
*matR*	Maturase R	92 082	A	C	K→Q	–0.223
ORF106	Hypothetical protein	25 588	C	A	H→N	–7.0
ORF295	Putative succinate dehydrogenase	25 760	C	A	H→N	0.6
ORF295	Putative succinate dehydrogenase	25 862	T	A	F→I	0.044
ORF295	Putative succinate dehydrogenase	25 863	T	A	F→Y	–0.103

### Comparison of legume organelle genome sequences

The coding regions of the faba bean chloroplast and mitochondrial genomes obtained from the resources provided by Sabir *et al.* (2014) and Negruk (2013) were compared with those of other legume organelle genomes that are available on the NCBI database (Benson *et al.*, 2013). Genome sequence alignment of 11 reference chloroplast sequences available for legumes (*Glycine max*, *Vicia faba*, *Lotus japonicus*, *Vigna unguiculata*, *L. culinaris*, *C. arietinum*, *Vigna radiata*, *P. sativum*; *M. truncatula*; *Arachis hypogaea*, and *Phaseolus vulgaris*) allowed for the identification of a core set of chloroplast genes that are conserved across all legumes (Fig. 5). Chloroplast sequences were well conserved (>80% for most genes encoding photosynthetic electron transport proteins). However, the percentage homology was lower in genes encoding ribosomal proteins. Between the plastid genomes, the greatest sequence variations were found in *clpP*, *ndhA*, and *rps12*, which showed <60% homology. The least variable genes were *psbE*, *psbF*, nd *psbD*, all of which had a homology >95%. The *psb* genes encode proteins that are part of PSII and therefore may be strongly conserved between species.

**Fig. 6.**
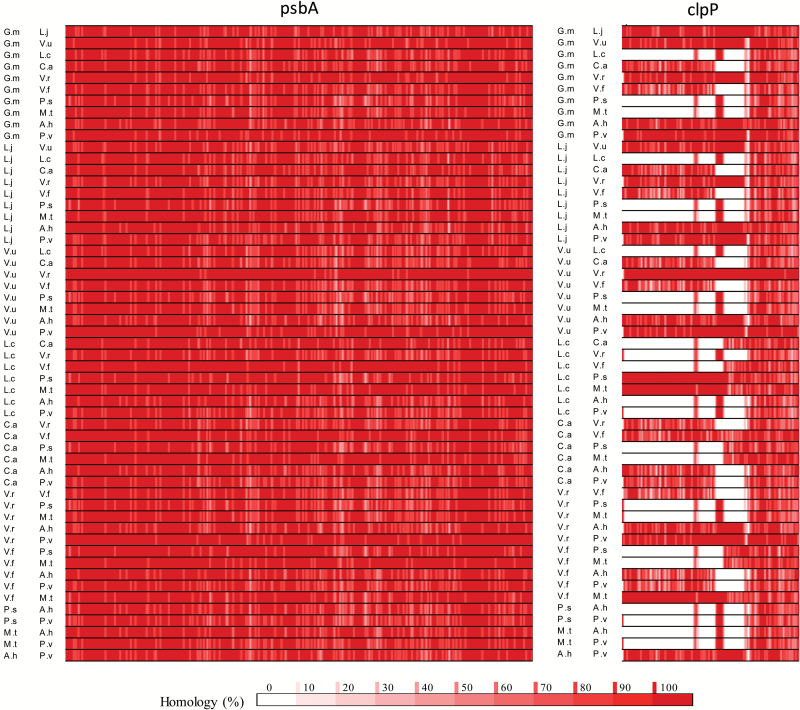
Representative examples of sequence identity maps drawn for the highly homologous *psbA* gene and the *clpP* gene with low homology. Maps were constructed from a multiway homology comparison between 11 legume species; *Glycine max* (G.m), *Lotus japonicus* (L.j), *Vigna unguiculata* (V.u), *Lens culinaris* (L.c), *Cicer arietinum* (C.a), *Vigna radiata* (V.r), *Pisum sativum* (P.s), *Medicago truncatula* (M.t), *Arachis hypogaea* (A.h), *Phaseolus vulgaris* (P.v), and *Vicia faba* (V.f). High degrees of homology between compared species are shown in dark red, while a lack of detectable matches is shown in white. Quantitation is as shown in the scale bar. Homology was determined across a 10 bp frame (i.e. a single polymorphism would give 90% homology).

**Fig. 7.**
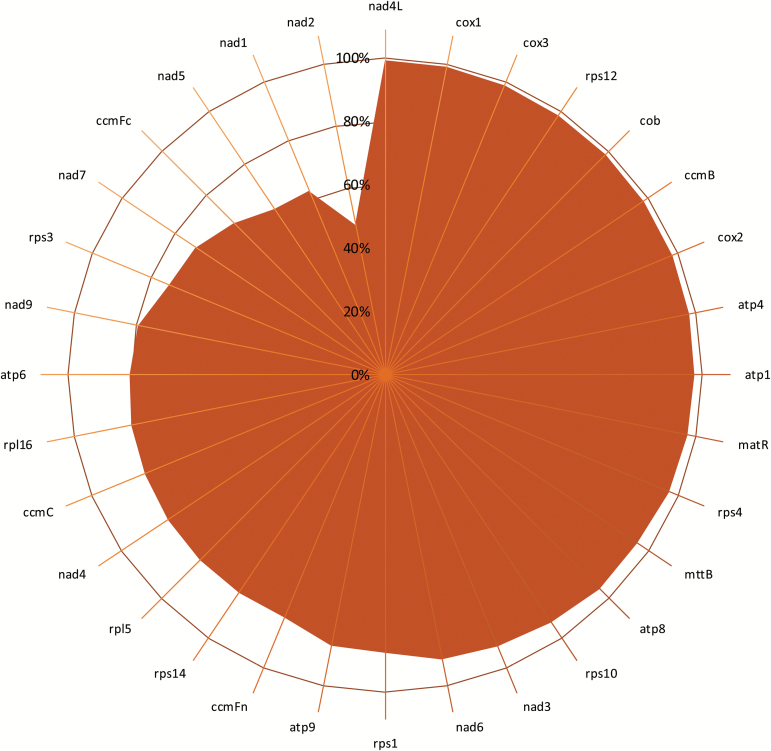
The percentage sequence identity for genes encoded in the mitochondrial genome. Average data for individual mitochondrial genes from six species were subjected to a 15-way alignment. Gene identities are given in the outermost circle. The scale shows the percentage sequence identity (0–100%). Species comparisons were performed for *Medicago truncatula* (M.t), *Lotus japonicus* (L.j), *Glycine max* (G.m), *Vigna angularis* (V.a), *Vigna radiata* (V.r), and *Vicia faba* (V.f).

Chloroplast genome variation was further analysed in homology maps in a pairwise manner for each gene and species. While *psbA* shows a very high homology across all species, *clpP* genes show far more interspecies variation, with large regions being absent from the coding sequences of *L. culinaris*, *M. truncatula*, and *P. sativum* (Fig. 6). Comparisons for all genes can be found in Supplementary Table S3. However, for simplicity, representative examples are shown in Fig. 7 for the *psbA* and *clpP* genes. 

**Fig. 8.**
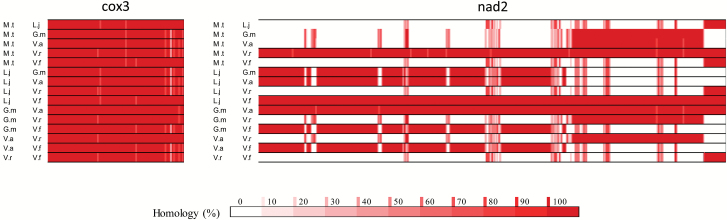
Percentage homology maps for *cox3* and *nad2*. Maps were constructed from a multiway homology comparison of six legume species: *Medicago truncatula* (M.t), *Lotus japonicus* (L.j), *Glycine max* (G.m), *Vigna angularis* (V.a), *Vigna radiata* (V.r), and *Vicia faba* (V.f). High degrees of homology are shown in dark red, and a lack of detectable match is shown in white, with quantitation as shown in the scale bar.

Reference mitochondrial genomes were available for six legume species on the NCBI database (Benson *et al.*, 2013). These sequences were compared to define a core set of conserved mitochondrial genes. Mitochondrial genome sequences are well conserved (Fig. 7), with all of the cytochrome-associated genes having an average sequence homology of >90%. However, genes encoding ribosomal proteins and the NADH dehydrogenase complex had a lower average sequence homology (Fig. 7). The most variable genes across the mitochondrial genomes were *nad5*, *nad1*, and *nad2* which showed <60% homology across the legume sequences analysed. The least variable genes were *nad4L*, *cox1*, and *cox3*, all of which had a homology >95%. Examples of the most (*nad2*) and least variable genes (*cox3*) are shown in Fig. 8.

## Discussion

While most plant species have small genomes (Kelly and Leitch, 2011), the data presented here confirm that faba bean belongs to those lineages that have ‘giant genomes’. This is rather surprising given that faba bean is diploid (2*n*=12 chromosomes). Chromosome fusion is known to have occurred during faba bean evolution (Fuchs *et al.*, 1998). As with other large genomes, genome size expansion can be accounted for by repetitive DNA sequences that consist of different types of transposable elements. In the data presented here, only 9.64% of reads could be identified in a repetitive element database (Bao *et al.*, 2015). However, many of the repetitive elements in the faba bean genome remain to be annotated and do not yet have a homologue in this database. Large plant nuclear genomes, such as that of maize, can contain 85% repetitive DNA made up of intact and simply organized retrotransposons (RTs; Schnable *et al.*, 2009). Many RTs that use element-encoded mRNAs as transposition intermediates can rapidly proliferate in copy number, resulting in large differences in genome sizes between related species. The action of these mobile elements can restructure the genome, resulting in a high level of genetic variability. Moreover, these RT-induced mutations are usually stable and can be used as molecular tools to study gene tagging and functional analysis (Bennetzen, 2000). The Gypsy and Copia type RTs belong to the LTR family and are referred to as Metaviridae and Pseudoviridae elements, respectively (Grandbastien, 2015). LTR replication via an RNA template is reminiscent of retroviral proliferation, and interestingly these are the predominant RTs in plants. Genome fluidity arising from the activities of these mobile elements has long been recognized as an opportunity for the genome to evolve in response to environmental challenges (Lisch, 2002; Wicker *et al.*, 2007). The expression of LTR RTs is important in host functional plasticity, with regulation being co-ordinated in response to a diverse array of external stresses (Reichmann *et al.*, 2012; Grandbastien, 2015; Giordani *et al.*, 2016).

RNA-seq data were used in the present study to identify a set of high confidence unigenes through alignment to the faba bean CSFL reference transcriptome. This analysis yielded a total of 16 300 unigenes with full depth coverage identified in CSFL. This subset was more likely to have an alignment to known genes than the full set of unigenes (71.2% versus 53.2%), but the alignment was no more likely to be longer (21.5 bp versus 21.0 bp) or to have a higher identity (82.2% versus 79.6%). The data were also more likely to yield fewer aligned DNA-seq read clips, indicative of transcriptome assembly breaks compared with the genomic architecture from which they originate. By combining the genomic sequence data presented here with the uncropped linkage marker data produced by Webb *et al.* (2016), sequence length data were increased at 461 chromosomal loci, extending current CDS sequence data into the genomic space.

Although the present characterization of the nuclear genome is challenging given the length and coverage of genomic reads, the mapping of the organellar genomes was much more informative. By comparing the plastome sequence reported here with the publicly available sequences (Sabir *et al.*, 2014), we identified relatively few variants in the coding regions. However, SNPs were identified in the genes encoding the RNA polymerase subunit C1 (*rpoC1*), the ATpase β subunit (*atpF*), glutamine tRNA (*trnQ-UUG*), and the β-carboxyl transferase subunit of acetyl co-A carboxylase (*accD*). Among these, *accD* contained the most SNPs, with three detectable polymorphisms. While the tobacco *accD* knockout mutants are lethal (Kode *et al.*, 2005), the *accD* gene was shown to be highly variable across 24 *M. truncatula* ecotypes (Csanad and Pal, 2014). Ecotype-specific *accD* gene sequences ranging from 650 to 796 amino acids in length were reported (Csanad and Pal, 2014). Despite this variability, a conserved carboxylase domain was always present, and gene length expansion and contraction was attributed to repetitive sequence integration in intronic regions (Csanad and Pal, 2014). The *accD* gene has been lost during evolution in a number of species (Rousseau-Gueutin *et al.*, 2013), but in such situations it has been functionally replaced by a nuclear-encoded analogue (Rousseau-Gueutin *et al.*, 2013).

Intraspecies comparisons of the faba bean chloroplast genes reported here may form the basis for future cultivar genotyping. A low level of plastome gene sequence variation was found amongst the 11 legumes analysed in the present study. There were notable exceptions however, particularly in the *rps2*, *rpl32*, *rps19*, *rps7*, *accD*, *rpl2*, *rpl23*, *clpP*, *ndhB*, *ndhA*, and *rps12* genes, which showed sequence homologies of only between 60% and 79%. These findings are consistent with the literature view that most photosynthesis-related genes are conserved to a higher level than ribosomal genes, with chloroplast genomes showing far higher degrees of conservation than the nuclear and mitochondrial genomes (Xu *et al.*, 2015). Despite the high level of variation in the *rps12* gene, loss of *rps12* function might be predicted to lead to impaired chloroplast translation (Ramundo *et al.*, 2013).

The number of polymorphisms in the mitochondrial genome was greater than those detected in the plastome, with six genes showing single base pair mutations compared with the reference sequence (Negruk, 2013). Two SNPs were identified in NADH dehydrogenase subunit 6 and maturase R, with ribosomal protein S14 and cytochrome *c* oxidase subunit II having six SNPs. In comparison with the chloroplasts, the mitochondria were found to be more prone to the accumulation of SNPs, although this was restricted to the intergenic spaces. Plant mitochondrial genomes are characterized by their very large size, ranging from 200 kb to 2500 kb, with many introns and repeated elements (Kitazaki *et al.*, 2010). Mitochondrial genome recombination is key to within- and between-species variation (Galtier, 2011). However, the rate of nucleotide substitution is low, mitochondrial genome variation within species being derived from dsDNA breakage and repair via highly efficient recombination of asymmetric sequences (Davila *et al.*, 2011).

In conclusion, the novel sequence data presented here provide an essential foundation for the generation of a draft nuclear genome sequence and further analysis of organellar genomes. The transcriptome reference for faba bean in the CSFL database is comprised of data from multiple cultivars. The data reported here for Wizard match this genomic resource but have advantages over the existing CSFL faba bean resource because they provide increased accuracy (Table 3). Given the rapid advances in sequencing technologies, it is feasible that a high quality faba bean reference genome will be produced in the near future by intense long-read sequencing, accompanied by innovative bioinformatics approaches. Unravelling the nuclear and organellar genomes of faba bean, their plasticity, and regulation in response to environmental challenges will not only increase our understanding of how stress tolerance is regulated but will also accelerate breeding progress. These data also provide an essential underpinning resource required for the genetic improvement of faba bean, an important cold season legume, paving the way for future realization of its considerable potential for improvement.

## Supplementary data

Supplementary data are available at *JXB* online.

Table S1. List of putative splice points identified by aligning DNA-seq reads to the CSFL.

Table S2. List of full length markers upon which the publication of Webb *et al.* (2016) was based, with aligned DNA contigs and sequence extension data.

Table S3. Pairwise alignments of sequences of identified genes in the chloroplast and mitochondrial genomes.

## Supplementary Material

supplementary_Table_S1

supplementary_Table_S2

supplementary_Table_S3
